# How to protect privacy in a datafied society? A presentation of multiple legal and conceptual approaches

**DOI:** 10.1007/s13347-022-00497-4

**Published:** 2022-01-29

**Authors:** Oskar J. Gstrein, Anne Beaulieu

**Affiliations:** Data Research Centre, Campus Fryslân, University of Groningen, Leeuwarden, The Netherlands

**Keywords:** Privacy, Datafication, Data Protection, Human Dignity, Technology

## Abstract

The United Nations confirmed that privacy remains a human right in the digital age, but our daily digital experiences and seemingly ever-increasing amounts of data suggest that privacy is a mundane, distributed and technologically mediated concept. This article explores privacy by mapping out different legal and conceptual approaches to privacy protection in the context of datafication. It provides an essential starting point to explore the entwinement of technological, ethical and regulatory dynamics. It clarifies why each of the presented approaches emphasises particular aspects and analyses the tensions that arise. The resulting overview provides insight into the main strengths and limitations of the different approaches arising from specific traditions. This analytic overview therefore serves as a key resource to analyse the usefulness of the approaches in the context of the increasing datafication of both private and public spheres.

Specifically, we contrast the approach focusing on data subjects whose data are being ‘protected’ with others, including Fair Information Practice Principles, the German right to ‘informational self-determination’, and the South American ‘habeas data’ doctrine. We also present and contrast emerging approaches to privacy (differential privacy, contextual integrity, group privacy) and discuss their intersection with datafication. In conclusion, we put forth that rather than aiming for one single solution that works worldwide and across all situations, it is essential to identify synergies and stumbling blocks between the various regulatory settings and newly emerging approaches.

## Living in the digital age

The relationships between collective and individual, between group and person, and between public and private, are fundamental to shaping our societies. These relationships are increasingly being played out in terms of data—its production, flow, reuse and commodification. Across the world, numerous different regulatory, scientific and societal frameworks have been used to govern and modulate these processes. For example, there are influential privacy laws or data protection regulations such as the European Union’s General Data Protection Regulation (GDPR) that shape who can collect which information for which purposes, and who has a say in this. Yet, it can be very difficult to understand why these different frameworks emphasise diverse aspects of privacy, how they define data, and what they precisely aim to protect—and from whom.

Furthermore, as platforms and other digital contexts increasingly have a global reach, the clash between different approaches becomes frequent. This article explores venues to the protection of privacy by mapping out and contrasting different legal and conceptual approaches in the context of datafication. It provides an essential starting point to explore the entwinement of technological, ethical and regulatory dynamics and serves as a key resource to analyse the usefulness of the different approaches. Therefore, it will appeal to users, designers, regulators and analysts who benefit from a concise overview of these different approaches and are interested in how they function in a datafied society.

Presenting and reflecting on the strengths and weaknesses of existing approaches seems all the more urgent since—according to the United Nations—we live in the ‘digital age’ (United Nations, 2013). This age is fuelled by ‘data’, which in 2006 was labelled as ‘the new oil’ by British mathematician Clive Humby (Arthur, 2013). While one may wonder whether such metaphors are helpful or not (Crawford, 2021, p. 113), data have become ‘big’ (Mayer-Schönberger & Cukier, 2014, pp. 19–32) and nearly omnipresent with the emergence of ‘smart infrastructure’ and digital platforms (Edwards, 2016; Ranchordás & Goanta, 2020). ‘Surveillance capitalism’, which exploits detailed consumer profiles of fully or partially personal information (Zuboff, 2019), is a central aspect for many economic activities. Digital data enable not only near-real time monitoring of aspects of human society (Galič et al., 2017, pp. 28–32), but also of nature (Rakhimberdiev et al., 2018), thanks to the increasing connection of data production, processing, modelling and sharing. Hence, digital data have become an important factor that (re-)shape the structures of our societies and environment. Although the power of the use of data is sometimes overestimated (Morozov, 2012, pp. 57–84), it is difficult to think of an area of life in the Global North that has not been subject to ‘datafication’ over the last decennia. As individuals and societies, we not only have to consider the value of our human labour, which is replaced by technology as artificial intelligence and autonomous systems evolve (Chelliah, 2017), but our individual and collective autonomy are also at stake as we become the subjects of ‘big nudging’ (Carolan, 2018).

These developments constitute new ways of being and make data deeply personal—changes that can seem unavoidable. Even if one does not care about datafication or deliberately objects to joining the ranks of billions of social network users all over the world, one can still be defined in relation to this datafication through ‘shadow profiles’; the large user base of big platforms makes it possible to deduce which non-users are connected to active social media users. Concretely, when users of most mainstream instant messaging apps (e.g. WhatsApp owned by Meta Platforms/Facebook) agree to the terms of service, this provides the company running mainstream apps with the entire content of the user’s address book, which also includes telephone numbers of non-users. From these data, not only the existence and identity, but also the kind of personal characteristics such non-users might have, can be extrapolated (Garcia, 2017). Hence, data generated by highly connected and monitored environments do not only allow one to make explicit statements about the individuals consenting to its collection and use, but also about those resisting it. The pervasive deployment of datafication also enables inferences on those who opt-out, regardless of how techniques such as anonymisation are being applied (Finck & Pallas, 2020, p. 35).

No one is an island in a ‘datafied’ society. Sharing the same time, space and cultural dimension makes it difficult for an individual to stand apart, especially if the societal architecture does not enable such objection (Cannataci, 2016). In parallel to what is being described as gentrification in cities where the wealth of one group deprives the less privileged of their opportunity to live autonomously and have a considerable stake in a social environment, we are witnessing ‘technological gentrification’ in environments that are permanently monitored and where those believing in the benefits of omnipresent data render the choices of others de-facto obsolete (Gstrein & Ritsema van Eck, 2018, p. 76). A complementary phenomenon is the cost of refusing to participate in regimes of datafication, a cost that becomes disproportionately high when very few individuals are in a position to object (Iacovou, 2019).

This observation leads to a first key point in this discussion of datafication and privacy: many insights about individuals rely on assumptions about populations and groups, whereas most privacy and data protection regimes focus on atomistic agents and their individual rights, often labelling them as autonomous individuals (Floridi, 2014). Some scholars try to fill this gap by exploring ‘group privacy’ (Taylor et al., 2017), demanding more democratic participation in processes of datafication (Viljoen, 2020), or by more broadly emphasising ethics and ‘data responsibility’. However, these efforts are in their infancy and much remains to be done to implement and enforce them in practice.

### Multiple legal and conceptual approaches

To better understand the tensions between approaches to the protection of privacy, the objective of this article is to concisely explain and analyse multiple approaches in the context of datafication. In a period where data are presented as neutral, unbiased and objectively evidential, it is especially important to analyse how datafication intersects with influential conceptions addressing privacy. This enables us to show commonalities and gaps in the way these concepts define data, as well as to explore their relationship to the subject, and to elaborate on the entwinement of technological, ethical and regulatory dynamics. While there is plenty of literature available that discusses all the concepts presented in this article in more depth, we identified a lack of studies that aim at exploring pathways towards synergy or friction between the various regulatory and newly emerging conceptual approaches.

It is important to make this variety of approaches towards the protection of privacy in a datafied society clearly visible for at least two reasons. First, analytically, contrasting different approaches to the protection of privacy throws into sharp relief the extent and way in which they differ. Second, it is important to make variation visible in the face of claims of the independence and universality of the Internet (Barlow, 1996). Given the lack of common standards and differing economic and political interests in digital networks and platforms (Aly et al., 2019; Griffiths, 2019; Gstrein, 2020a, pp. 1–3), in combination with the seeming ubiquity of data-driven applications for managing identity (Zwitter et al., 2020), it is crucial to understand how data technologies can have a variety of implications for privacy.

To be able to carry out this mapping exercise, we choose to focus on contrasting concepts that address the protection of privacy in a datafied society. This allows to illustrate the variety of approaches and their implications. From the outset, we focus on legal approaches with the objective to protect the private sphere and personal data. They are particularly relevant due to their formally binding nature. Their function in establishing rights and duties in society gives them considerable authority, often gained throughout a history of political struggle and societal discourse in many regions worldwide. Therefore, they enshrine long-standing traditions and beliefs. Specifically, we consider the development of privacy as an individual right that is globally protected in the form of international human rights law today, as well as the development of data protection regimes from the 1970s onwards and which in the year 2021 exist in 145 countries worldwide (Greenleaf, 2021). While these regulatory ‘mainstream approaches’ have their own problems when defining what privacy is and why it should be protected (González Fuster, 2014; Purtova, 2018; van der Sloot, 2014), we further enhance the review of regulatory concepts with two alternative frameworks from South America and Germany. In a next step, we add to this regulatory overview by presenting recently emerging approaches to the protection of privacy from different areas. While these are not as established as the regulatory regimes, they might inform the development of future governance frameworks and of measures that promote the protection of privacy in the context of datafication.

Certainly, our selection of approaches to the protection of privacy in a datafied society is limited and partial. Scores of other related approaches, such as confidentiality, anonymity and even intellectual property, could have been considered since they can all shape what we consider to be ‘ours’ or what should be ‘out-of-bounds’ when it comes to use and circulation of data. Additionally, while we strive for the presentation of a wide geographic spectrum, approaches of indigenous communities to data sovereignty (Carroll et al., 2019), as well as the perspective of non-Western philosophical traditions such as the African Ubuntu, could have been considered in more depth (Reviglio & Alunge, 2020). Finally, novel conceptualisations of privacy in the context of datafication emerge continuously (see e.g. Søe, 2021). Our selection strives to provide an initial mapping as a starting point for other scholars and students. Within the limited length of an article, we narrowed down our selection to combine a diverse range of concepts and their discussion at an appropriate level of detail for non-legal specialists.

## Datafication

We begin by explaining how datafication forms a specific context for concepts of privacy and their articulation. Datafication is a complex process that is often associated with digital technologies and (Big) data infrastructure. Furthermore, data and data-related capabilities are central to datafication. This includes the extension of automation, the proliferation of digital technologies, the willing production of massive amounts of data and the combination and circulation of datasets (Rieder & Simon, 2016). Datafication relies on the spread of connected digital devices and on infrastructures made up of networks and platforms. Nevertheless, technological possibilities alone are not sufficient to explain the scope of datafication; societal practices and configurations must also be considered (Beaulieu & Leonelli, 2021).

From this perspective, datafication is the understanding of objects and processes as data. As Van Dijck and colleagues show, this ‘turning into data’ has several dimensions (Dijck et al., 2018). Networked platforms turn many aspects of the world, such as social ties and connections, into digital traces. Our ‘friends’ on Facebook, or the accounts we follow on Twitter, are recorded as digital data. Datafication is also the process of rendering such relations and activities on platforms as quantifiable traces in which behavioural patterns can be discovered. For example, the platform LinkedIn makes it possible to create an individual profile, including a photograph, and to list one’s employment history. LinkedIn keeps track of when users update their profiles and when they change their employment data. The company has identified patterns of activity on users’ profiles (updating your profile photograph) that indicate that an account holder is likely looking for a job and is therefore a good target for job ads. The use of algorithms in hiring has also been proliferating (Leicht-Deobald et al. 2019). Furthermore, datafication is the transformation of interactions into data that can be valued and used for predictive activities (Beaulieu & Leonelli, 2021). Examples of this are the analysis of public sentiment via social networks, as well as using targeted advertising to influence and predict electoral outcomes (Coppock et al., 2020). Another timely example is the tracking of population movements via telecommunication data or localisation of smartphones, and its use to predict and prevent the spread of COVID-19 (Zwitter & Gstrein, 2020a, b, p. 2). Finally, datafication is the extension of the collection of traces of every interaction. This ‘recording by default’ is enabled by omnipresent sensors and cheap storage, which creates the effect that the labour required for data selection is comparatively costly (Mayer-Schönberger, 2011, pp. 50–92). Although some of these randomly collected data are not meaningful from the outset, it can still lead to results eventually. An instance of this is the discovery and use of surprising correlations, such as the relation between a user’s typing speed and depression (Mastoras et al., 2019).

Given the scope of datafication, it is not surprising that it intersects with the concepts, structures and practices that are used to define and protect privacy. Datafication affects the circulation of data. It makes visible and traceable intimate, personal behaviours and generates traces of the functioning of our bodies. Simultaneously, datafication expands the rate and types of communication in which we engage. These are all terrains where privacy has been formulated and regulated. Before moving to a discussion of how datafication challenges privacy, we delve deeper into datafication as a layered practice and provide an explicit model of the central aspects of datafication. This model is useful in understanding the complex challenges to the approaches presented in Sect. 3.

### Central Aspects of Datafication

**Fig.1:**
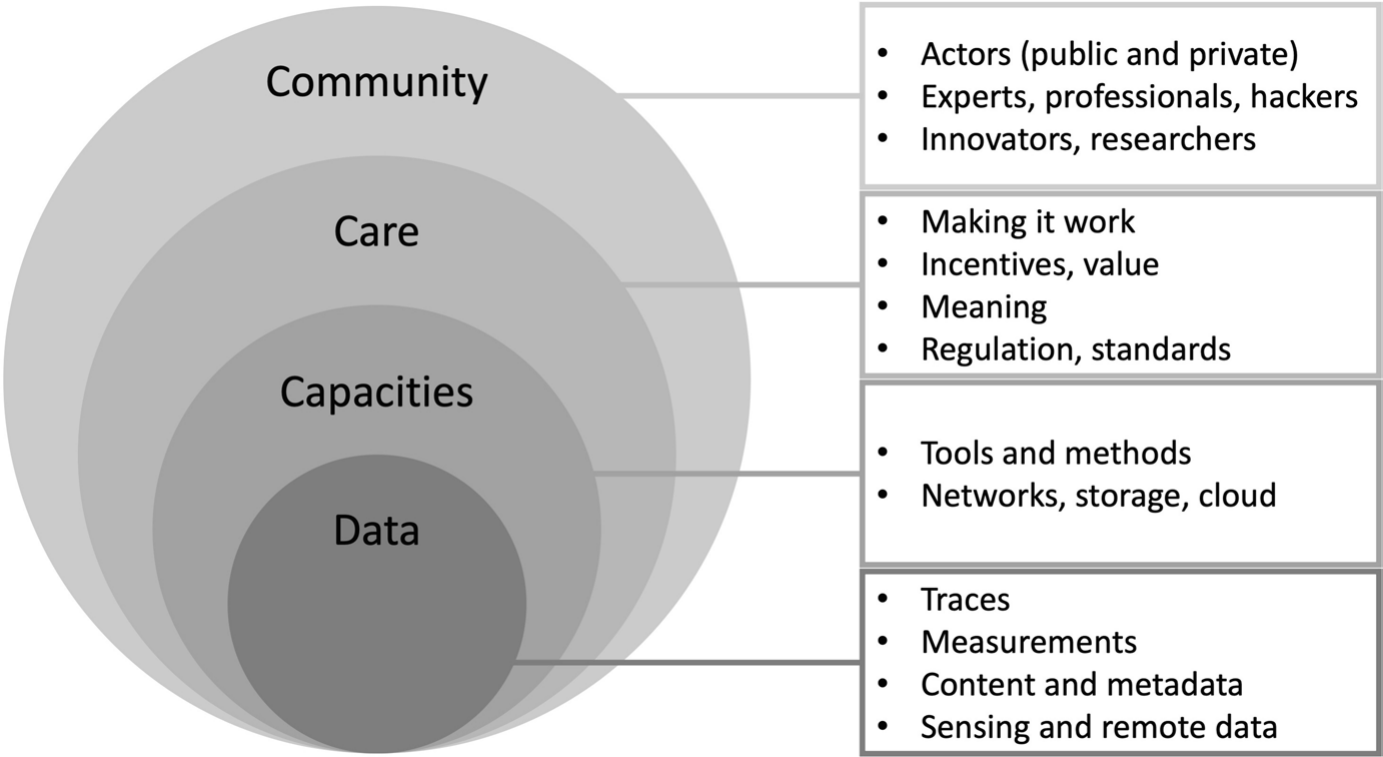
The layers of datafication. This graphic is adapted from the ecosystem of Big Data (Letouze, 2015) through the addition of the care sphere and a conceptualisation of the meaning of each layer. Reproduced from (Beaulieu & Leonelli, 2021)

As soon as one considers the various environments and practices involved in making and interpreting data, it becomes clear that datafication is not only about data and related computational techniques. For this reason, Beaulieu and Leonelli (2021) propose a layered model that puts neither data nor technology at its centre. Accordingly, datafication is a practice that links four essential elements:

- First, the community of actors (and related institutions) who engage with the data. For example, those who handle data on an everyday basis—to use, innovate or challenge it. Such communities can be more or less homogeneous and distributed.
- Second, the forms of care for data. These include how meaning is attributed to data, how it is valued, and the regulations and laws that shape the use of data. This layer also includes the work done to care for data and to maintain, repair or preserve it.
- Third, the capacities needed to handle data. These capacities can be those of humans (skills, training and experience) or technologies (storage and distribution) and techniques (statistical methods, computational tools, machine algorithms).
- Fourth, data, which can take many forms (numbers, images, text, symbols, sound). Data can also be more or less intentionally and explicitly generated, as part of measurement or experiments or gathered in more ad hoc ways (data exhaust) (Fig. 1).

This layered understanding of datafication makes clear that neither the challenges nor their solutions are simple since the layers are in contact and feedback loops can arise. If we understand datafication as being composed of these four layers, then we cannot hope to regulate datafication by simply intervening in a set of technologies or a specific sector. For example, intervening in the circulation of data would not be effective unless the capacities and the community are also aligned with data requirements. Similarly, understanding the different layers of datafication shows the shortcomings of approaches that focus solely on individual rights without consideration of effects at the level of community.

### Datafication and privacy

Before delving into the analysis of specific concepts (Sect. 4), we consider several ways in which datafication changes the very grounds from which privacy-related concepts arise. Datafication is an intense and prominent phenomenon since the first decades of the twenty-first century and poses many challenges to our understanding of privacy—or what is ‘personal’. However, it is not the first time that there is a reconfiguration of the elements that underlie our understanding of privacy. At different times, novel constellations of technologies, meanings and practices have led to shifts in boundaries between what is considered public or private, social, secret or intimate spaces. While it is sometimes stated that the current discussion around privacy is revolutionary, new or unique, in-depth studies on the establishment of private, family and public spaces in many societies and different historic periods suggest otherwise.

Among the plentiful literature on this topic, we highlight two studies to briefly illustrate this point about historical and cultural variation. First, Stanner describes how privacy-related behaviour can be witnessed in aboriginal settlements in Australia, despite seemingly constituting a ‘*community of the eye*’ where all actions take place in the absence of brick walls or windows. He refers to the fieldwork of anthropologists documenting cases of insensitive intrusion in intimate and private spaces in aboriginal camps, enforced over-crowding on an unprecedented scale, arbitrary acts of authority, public disparagement of precious goods with emotional value, as well as the commercial or private exploitation of goods by European colonisers, who neglected their essential role in the conception of the self in Aboriginal philosophy (Stanner & Martin, 2001, pp. 152–153). Secondly, a study that points in a similar direction describes the development of privacy-related case law in Aleppo, Syria, throughout the eighteenth century. The cases described in this study were collected by the Syrian authorities in court chronicles. They relate to privacy in a physical and informational context, usually described through terms such as modesty, sexual morality, civility, respect, honour and other societal values. Frequently, there is a connection to the religion of Islam and established social practices (Marcus, 1986). While a contemporary observer might find some of the aspects and subjects of these case studies more relatable than others, these descriptions of changing societal configurations highlight that privacy is a dynamic concept with a long history and the tendency to constantly transform. In this article, we consider how this transformation takes place through datafication, which currently reshapes privacy and its constitutive elements. We note the importance of the performance of privacy, the increased mediation and visibility of our bodies and behaviours, as well as the growth in the type and intensity of communication and circulation of data.

Datafication transforms our way of ‘being a person’ and enjoying privacy in many ways. Most obviously, there is a proliferation of techniques and practices that shape our profiles and identities that ensure our access to all kinds of digital spaces and services. Datafication of our persons takes place through various systems that authenticate our identity, allowing us to exert our rights (voting), to fulfil our duties (filing an income tax return) or benefiting from our rights (enjoying family life, child benefits, housing subsidies). Therefore, we can speak of ‘datafied identities’ as forms of personhood (Turkle, 1996). In the early years of widespread internet use, there was a pervasive celebration of multiple identities, fluidity, play and of virtual embodiments that were separate from ‘meat’ personhood. However, with the introduction of requirements to use email to validate registrations, the multiplicity started to be funnelled into a narrower set of possibilities due to requirements for verification. With Web 2.0 came the strong nudge to fill in ‘profiles’ to further detail and verify identities. Most recently, we are seeing a push towards integration, convergence and connection of identities (for example, signing in with Google or Facebook on multiple platforms, also discussed below). Besides the power of forms of authentication of our identities, datafication also affects how we perform our identities. In presenting ourselves to others in highly datafied contexts, we pay attention to what data might say about us by managing our social media profiles. As users of social media, we post information in particular ways that we hope are likely to yield the kind of profile and garner the kind of attention we consider desirable. In doing this, we take into account what we know about how algorithms and platforms work and are aware of the (limited) control we have on how audiences perceive us (Beaulieu & Estalella, 2012; Pitcan et al., 2018). These changes cannot simply be attributed to the adoption of digital technologies—they are the result of datafication. Identity, access and self-presentation are all elements that matter for what counts as personal, private or intimate.

Datafication is also relevant for increasing the terrain that might be relevant for privacy. The use of social media and dating apps has created traces of our social interactions. Datafication also leads to increased mediation of our bodies and behaviours. It creates traces of what was previously not only very personal and intimate, but also fleeting. Datafication turns these into data that can be collected, as well as circulated and shared. The area of biometrics has hugely increased the use of traces that are closely tied to our bodies. Datafication can also connect earning a livelihood and legal identity in ways that may be detrimental to individuals (Gstrein & Kochenov, 2021, pp. 72–76). For example, when undocumented immigrants become more visible because they use digital platforms to find work, they may be more easily exposed to surveillance and detection by immigration authorities (Ticona, 2016). Datafication can create new possibilities—think of quick border checks, detection of illegal migration at coastal areas using facial recognition or more effective management of refugee camps via iris scans—but it can also lead to new vulnerabilities for which existing privacy laws might not offer protection.

Yet another intersection of datafication and privacy has to do with the changing scope and intensity of communication. The personal and public spheres are commonly used as foundational concepts for privacy, as we will see in our review below that expands on the protection of privacy as an individual right, the parallel emergence of data protection in the twentieth century and other legal approaches such as habeas data and informational self-determination. What can be communicated across spheres and what should remain in the private sphere have long been at the heart of debates about proper use of communication media. In an important piece by Warren and Brandeis from 1890, which is sometimes lauded as the origin of the modern privacy discourse, the authors complain: ‘[i]nstantaneous photographs and news-paper enterprise have invaded the sacred precincts of private and domestic life; and numerous mechanical devices threaten to make good the prediction that “what is whispered in the closet shall be proclaimed from the house-top”’ (Warren & Brandeis, 1890, p. 195).

Rumour has it that the authors drew the motivation for their work from frustration about the public reporting of a family wedding in a period where photography became more popular and in which newspapers reported more broadly on social events. Whether this is true or not is subject to academic dispute, but it seems likely that without the unexpectedly broad press for the wedding, this famous article would not have been written (Gajda, 2007, pp. 36–42). One can only speculate how Warren and Brandeis would react to social networks such as Instagram and related discussions of the regulation of ‘influencers’ (Goanta & Ranchordás, 2020). New practices, in which for example photographs might not only be published, but also tagged, liked, shared or turned into memes, change how we think about and experience the private and public spheres. Also complicating the relation between spheres is that they are increasingly populated by different types of agents. By using sensors and algorithms, situations arise where data are processed by machines, rather than viewed or read by humans. This raises the issue of whether the access to data by a machine is as sensitive as access to data by a human, again challenging the usual categories on which privacy is articulated. For instance, the GDPR gives the individual a right to claim a human review of automated decisions in Article 22, but it is still unclear whether this is practicable and effective (Wachter & Mittelstadt, 2019; Wachter et al., 2017).

Finally, datafication has greatly increased the circulation and processing of data, which is typically regulated through user agreements with a platform owner or service provider (see especially Sects. 4.1.1. and 4.1.2). A significant trend has been the provision of services on one platform, based on a profile from another platform—for example, logging on to LinkedIn using a Google profile. These possibilities are presented as adding convenience for the user. However, such practices muddle which ‘agreements’ are in place (e.g. that of the platform being signed on to or that of the platform used for identification). The connection of different accounts enriches the profiles available to all platform owners, since they combine different types of information from different activities (for example, searching and shopping, or emailing and professional networking). The enriched profiles thus created have become important commodities (Beaulieu & Leonelli, 2021; Zuboff, 2019). In addition, these linkages across platforms narrow the possibility of being different types of persons in different contexts since authentication from a single profile is implemented across different contexts. Possession—or lack—of digitally verifiable credentials therefore translates personal data into economic value, shapes who we can be on the internet, and connects the private and the public in new ways. (On the internet, platforms know exactly what kind of dog you are and which segment of dogfood they can sell you.) Potentially, these many changes brought about through datafication can affect individual and collective self-determination, as well as human dignity (Zwitter et al., 2020, pp. 10–12).

## Approaches to the protection of privacy

While this larger terrain is deserving of critical examination, we focus here on the intersection of datafication and approaches used to articulate privacy and its protection. We map out several legal and newly emerging approaches, with the aim to contribute to clarifying the heterogeneous elements that make it difficult to debate, regulate and protect privacy in the context of datafication. We connect our analysis of forms of privacy to specific instances of datafication, so that instances ‘stand in’ as exemplars for different approaches (see Table 1). We contrast and compare different concepts across contexts, so that their particularity comes into relief and provides insight into the tensions and overlaps in regulating privacy.

**Table 1:** Summary of concepts and instances relating to privacy and datafication. This table supplements the detailed discussion in this article and provides a concise overview that highlights the contrast and overlap between different approaches

Legal approaches ﻿(Sect. 4.1)	Conceptualisation of Privacy	Instance of datafication
Privacy (US) 4^th^ amendment focused	Right to be left alone: protection from warrantless search; establishes realm of the personal by excluding it from public scrutiny (effectively scrutiny from governmental powers); realm of the private is defined by ‘reasonable expectation of privacy’ afforded only to US residents/’persons’—all other humans are without protection (for example, immigrants at border); 4^th^ Amendment only regulates interactions between government and citizens First established through Seymane’s case (1604) 5 Coke Rep. 91; protection of home against abuse of power by government through jurisprudence; ‘knock-and-announce’ rule; ‘my home is my castle’	GPS placed on car by law enforcement agencies: violates reasonable expectation of privacy (unauthorised GPS monitoring (U.S. v. Jones) Call Data Record (Verizon) used to prove involvement in armed robberies; Carpenter vs. USA 2015 San Bernardino terrorist attacks: In the aftermath, US authorities asked Apple to provide information to crack suspect’s phone; Apple refused, but phone still cracked with support of an Israeli firm, thereby showing need for protection of users from government intervention
Fair Information Practice Principles, FIPPs (US)	A set of 8 principles developed in the USA in the 1960s to regulate relations between customers and companies. Two principles are especially important: notice and choice Regulation traditionally applying a ‘sectoral approach’. Legal/Regulatory frameworks can be very detailed and practical, but sometimes non-existent or difficult to keep up to date due to speed of technological innovation Generally, data flow freely unless restricted in a specific instance for a specific sector. Regulation of business activities only applies if there is commercial agreement between business and customer. In the USA, divided among federal and state contract law, general and sector-specific consumer protection laws (e.g. the Health Insurance Portability and Accountability Act, the Children’s Online Privacy and Protection Act) apply, as well as tort law	Notice has taken the form of very extensive texts written in complex legal terms presented as a pop-up window, while choice has been reduced to ‘clicking’ a box labelled ‘accept’. To what extent ‘notice’ in this form is acceptable and whether it is actually possible to choose are both debated, since the cost of exclusion from digital services can be so high as to make it nearly impossible for an individual to choose to refuse, if it means exclusion from employment, education or health care
Data protection (EU, Council of Europe)	Comprehensive regulatory frameworks (‘omnibus approach’) based on principles such as data minimisation and purpose limitation No use (flow) of data without specific legal basis. Individual consent is frequently used, but such a basis might also originate from public interest (e.g. research, keeping statistical record), or prevailing interest of other individuals Fundamental right (next to traditional privacy right) in the EU; the existence as an additional right is potentially relevant in areas where data are combined to generate inferences on individual behaviour/characteristics and to be able to make a case about impact of data on groups Three key parties: data subject (= identifiable natural person depending on interpretation of abstract/concrete criterion); controller (main responsibility); data processor (technical/organisational support) Includes many individual rights such as the right to know about collection, the right to request one’s data and to be forgotten; the right to data portability; the right to review of automated decisions, etc First laws since early 1970s, developed since then continuously on national, international (e.g. Council of Europe Convention 108) and supranational levels (e.g. EU GDPR)	The ‘right to be forgotten’ can be implemented as the right of a person to have a link relating to their name which is inaccurate, misleading, or distressing removed from the search engine result. This would potentially also provide recourse in face of malicious activities like ‘revenge porn’ When a European citizen is the subject of a decision made by an algorithmically driven system, they are entitled to review of such a decision. For example, Uber’s use of an automated fraud detection system led to firing of drivers. An Amsterdam court ruled that this practice contravenes Article 22 of the EU General Data Protection Regulation (GDPR), which seeks to protect individuals from automated decision-making. Uber was ordered to reinstate the drivers, with compensation
Habeas data (South America, Argentina and Brazil)	Literally means ‘the data belongs to the body’; reaction to the political situation in dictatorships in South America, but not always clearly defined conceptually Narrowly understood, it entitles a citizen to obtain all available information about oneself (or close family members only), specifically in the context of a court/administrative procedure Broadly understood, it enables anyone to access information from public archives, and potentially also from private sources in cases where this information might be of public interest Established as a legal right in the constitutions of several Latin American countries including Brazil and Argentina	Getting access to data of ‘disappeared persons’ (desaparecidos) in the aftermath of Argentinian military dictatorship (1976–1983). The relevant data are typically held in public archives, but there is no clear possibility or institutional mechanism that provides access for family members, researchers, or the public without ‘habeas data’. While the right is usually acknowledged for public archives, there are doubts whether private sources are also included
Informational self-determination (Germany)	Based on ‘right to personal development’, derived from the German constitution through a combination of the protection of ‘human dignity’ and general right to personality (‘allgemeines Persönlichkeitsrecht’) Established by the German Federal Constitutional Court in the ‘census judgment’ from 1983 (BVerfG, ‘Volkszählungsurteil’, 15.1.1983 – 1 BvR 209/83, 269/83, 362/83, 420/83, 440/83, 484/83, BVerfGE 65, 1) Right to decide which information about self is to be communicated; Protection from unlimited collection, use and storage of data Not being afraid of what the state knows or might know; room for self-development—strongly connected to view of life as divided up into public and private spheres The person is an actor who decides and can stop data collection; state-citizen relationship is the focus	Privacy notices on a website are meant to enable voluntary, specific, informed, and unambiguous consent of individuals to particular uses of their data, thereby enabling informational self-determination. But what does it mean to accept such complex and lengthy notices? Are such practices to express consent functional and sufficiently transparent? This raises the issue of whether someone is making an informed decision and whether one has a choice in the matter A regional academic hospital carries out a large-scale longitudinal study through which it collects genetic material and keeps health records of families. The original intention is to understand genetic and hereditary diseases better to improve public health. Once the data are collected and included in studies (publications and research), can the individuals from whom the data were collected still determine what happens to it? How can it be guaranteed they will not have to be concerned about how public and private institutions use insights derived from their data?
Emerging conceptual approaches (Sect. 4.2)		
Differential privacy (in different jurisdictions, in relation to the privacy-preserving creation of statistical insights)	A statistical/mathematical method to make it more difficult to look up or infer personally identifiable information from large datasets. Artificial ‘noise’ is added to data collected on individuals. This makes it difficult to analyse individual data points, while significant overall trends (‘population level insights’) across the entire sample remain visible Three important parameters for implementation: -accuracy of the dataset -artificial adjustments making the set less accurate on a granular level (noise) -number of queries available to probe the dataset (‘privacy budget’) Proponents claim it adds privacy protection ‘by default’ while enabling the responsible use of large datasets. However, the method is difficult to implement throughout an entire process when many actors are involved and requires a high level of data literacy to be able to interpret datasets correctly. Additionally, it is not useful in cases where accurate data on individuals are required for analysis and processing	A globally active technology corporation wants to understand how a new app is being used in a certain market and whether available features are being picked up by users or not. The insights on an individual level are not so important as the overall picture Policymakers want to understand whether a curfew leads to less mobility across the entire population of a country using search queries or mobile phone data, to determine whether citizens/residents are actually staying at home (e.g. COVID-19 movement restrictions)
Contextual integrity (USA in origin, not applied as formally binding concept)	Starting point is expectations of privacy—distributed across actants (persons and technologies) Actants will have different privacy expectations based on context and flow; data flows and systems are designed accordingly and with appropriate levels of safeguards depending on context Parameters of informational norms are actors (subject, sender, recipient), attributes (types of information), and transmission principles (constraints under which information flows); against ‘transparency and choice’ approach because it is not context-sensitive	Apparently harmless data (how often you log on and at what time of the day) get associated with your profile and is used to target you (you must be lonely and insecure, here is an advertisement for a new leather jacket). In this example, there is a shift in context from social platform to marketing opportunity
Group Privacy (in different jurisdictions, in reaction to individualisation of privacy)	Looking at how the use of data impacts groups and their collective autonomy for future development ‘Big Data’-driven applications might not directly impact individual or do so based on individual consent, but the insights created by them reshape society Difficult to put in practice since it is largely unclear who should take agency on behalf of whom	Predictive Policing; predicting the likelihood of crime based on statistical methods and different sources of open/closed data, often based on historical data for a given area Using algorithm-based systems to create profiles for selecting applicants for job interviews

### Legal approaches

#### Privacy as an individual right

Privacy is a universal human right, enshrined in Article 12 of the United Nations Universal Declaration of Human Rights from 1948, as well as Article 17 of the International Covenant on Civil and Political Rights from 1966. Although the wording and structure of both provisions are very similar, only the latter document was drafted from inception to become legally binding for states to protect the rights of individuals (Joseph & Castan, 2013, pp. 160–164). While Europe is particularly rich when it comes to formal guarantees—with international treaties such as the European Convention on Human Rights (ECHR) or the Charter of Fundamental Rights of the European Union (CFEU) that are mutually reenforcing (Polakiewicz, 2015)—similar standards exist in all regions of the world. These have been compiled in a list by the United Nations Special Rapporteur on the right to privacy (United Nations, 2020). Besides relevant international treaties and guarantees, it is useful to explore the emergence of privacy as a human right. Privacy is a formally guaranteed civil right in national constitutions such as the Bill of Rights of the USA. With regard to the US context, the fourth amendment from 1790 is of particular interest. It is among the first attempts to formalise the protection of private space and individual autonomy. This historical relevance and the fact that it is still in force today highlight the importance for defining privacy and allow to illustrate the consequences of datafication.

The fourth amendment refers to physical search and seizure by government in private premises. It has roots in English case law scrutinising enforcement of general warrants and writs of assistance, which was often deemed to be too invasive. Among this jurisprudence is Seymane’s case from 1604 in which the famous English lawyer Sir Edward Coke provided the groundwork for what later would become the proverb ‘my home is my castle’. This issue of protection from government interference in private premises was especially problematic for individuals living in colonies of the English crown, since they had less protection against governmental abuse of powers than those in the ‘homeland’ (Gray, 2017, pp. 69–72). Hence, when a Bill of Rights was drafted to augment the new US constitution, a safeguard against governmental search and seizure was included, based on this historical experience.

The amendment articulates the traditional substantive scope of privacy protection that includes personal (physical) space, family life, as well as the protection of honour. The related right to confidentiality of communication (e.g. through letters) later emerged, although this development also has to be considered in the context of the development of freedom of expression (Borgesius & Steenbruggen, 2019, pp. 293–297). Privacy should therefore not be understood as a siloed topic. Rather, it is a proxy for the relationship between the individual and society and also needs to include consideration of freedom of expression, accessibility of information and further elements as they evolve dynamically along the vectors of time, space and culture (Cannataci, 2016, pp. 8–10).

The ‘traditional’ distinction between private and public spheres and their correspondence to differentiated physical private and public spaces has been challenged by social and technological developments. The emergence of telecommunication in the twentieth century and the process of datafication in the twenty-first century have made it increasingly difficult to interpret and apply traditional privacy norms. Clearly, the dynamics of datafication that redraw the private and the public—separating our understanding from the physical space of the home—further challenge this understanding of privacy. In the famous 1967 case of Katz vs USA, the US Supreme Court had to decide whether the use of an ‘electronic ear’ to eavesdrop on illegal sports betting in a public phone booth violated ‘reasonable expectations of privacy’ (Gray, 2017, pp. 76–89). This sparked decades of American jurisprudence through which more and more abstraction of the historical context and meaning of the law occurred. Both what it means to gain access and what constitutes private premises are affected by datafication. In terms of access, ‘spaces’ are not entered through physical force (e.g. kicking down a door) but through exploitation of security vulnerabilities in information systems (e.g. accessing a smartphone with spyware). The latter are sometimes even mandated by democratically elected governments that enforce mediocre security and encryption standards for devices and cloud storage services, so that they can potentially be more easily accessed (Mann et al., 2020; Schneier, 2019, pp. 13–16).

Another example of the reworking of public and private can be found in Hildebrandt, who draws on Agre’s work: whereas a camera might be used for surveillance purposes—a technology entering a sphere of life and intruding on privacy—GSM technology makes it possible to ‘view’ an individual’s movements because the environment has been reconfigured by sensor and networked technologies to capture a person’s movements (Hildebrandt, 2019, pp. 89–95). Related case law on the use of geolocation devices for investigative purposes exists in the USA (e.g. USA vs. Jones (No. 10–1259, 565 U.S. 400) from 2012), but also in Europe on the level of the European Court of Human Rights in Strasbourg (e.g. 2010 case of Uzun vs. Germany—application no. 35623/05—and 2018 case of Ben Faiza vs. France—application no. 31446/12). The use of data permanently produced, shared and recorded by omnipresent devices and networks continues to pose challenging questions, especially since the division of regulation of datafication processes in the public and private sector becomes increasingly difficult. This can briefly be illustrated with three examples.

First, in the 2018 landmark ruling of the Supreme Court in Carpenter vs. USA (No. 16–402, 585 U.S. (2018)) the key question was whether the records of the movement of a cell phone between cell towers constituted private or public data. These records were originally intended for billing purposes, before being repurposed as essential evidence in this criminal procedure. The judges ultimately decided in a very controversial case that the records belong to the private domain of Carpenter, which meant that they could not be used for criminal investigations by law enforcement, although Carpenter’s involvement in criminal activities seemed likely (Klein, 2019). A second example can be found in the successful identification of the ‘Golden State Killer’ following an investigation spanning decades that was only possible with the emergence of privately owned and loosely regulated data infrastructure to process and match human genetic data (Levenson, 2020). Finally, the case of ‘Clearview AI’ demonstrates how privately held data scraped from websites can be used to enable facial recognition on population level. The company behind the service offers access to a large database built on image data scraped from social media websites and other platforms with photographs of individuals. Clearview allows the matching of footage from surveillance cameras and other sources with the information available on the internet. Access is available for whoever books the service, which seems especially appealing in a law enforcement context in the USA (Rezende, 2020). Similar services with huge databases containing images of people’s faces emerged in Poland (‘PimEyes’) and in the Russian Federation (‘FindFace’) (Brewster, 2020; Laufer, 2020).

To widen the area of consideration even further, in the late 1960s, around the same time the Supreme Court was deciding the Katz case, the ‘Fair Information Practice Principles’ (FIPPs) were developed in the USA. They form the basis of US regulation and policies addressing the use of personally identifiable information (PII) (Solove & Hartzog, 2014, pp. 593–595). These eight principles became very influential for the regulation of data-driven activities, especially in the form of the US Privacy Act of 1974 (Hoofnagle et al., 2019, p. 70; Rustad & Koenig, 2019, pp. 411–419). However, contemporary US privacy law for the private sector lacks a consistent approach that covers all kinds of data-driven activities benefitting from the use of PII. This lack of an effective and politically accepted conceptual embedding for privacy protection leads to frequent attempts to re-label the subject as ‘data privacy’, ‘online privacy’ or ‘information privacy’. Currently existing law comprises federal and state contract law, general and sector-specific consumer protection laws (e.g. the Health Insurance Portability and Accountability Act, the Children’s Online Privacy and Protection Act), as well as tort law (Rustad & Koenig, 2019, pp. 381–387; Viljoen, 2020, pp. 592-597). In regulating the activities of businesses, two of the FIPPs are most prominent: notice and choice. This means that consumers entering a relationship with a business should be given notice of the business’ information practices before personal information is collected, and that this notice forms the basis on which a customer can make an informed choice about whether to disclose PII. Choice can take an opt-in or opt-out form.

The model of datafication presented above helps to tease apart the complex transformations that give rise to the challenges reviewed in these examples. In the case where a private space is infused with data and new capacities for accessing these data are available, this leads to the question how access should be understood from a privacy preserving perspective. Similarly, when participation in a community relies on agreement to the generation and circulation of data, to what extent can the right to privacy be effectively protected? Furthermore, these principles may fall short of protecting privacy in the context of datafication and of the growing significance of the economic value of data-driven business. There seems to be increasing consensus that the ‘notice and choice’ model largely fails to address potential harms, which do not only relate to individual data subjects (Bradford, 2020, pp. 140–141; Viljoen, 2020, pp. 597-603). Furthermore, the likelihood of truly understanding notice has been questioned, as the documents outlining general terms and conditions become increasingly complex, lengthy and are frequently updated (Beaulieu & Leonelli, 2021). In addition, choice may be highly artificial when refusal comes at a very high cost for individuals. For instance, it might not even be viable to expect casual news readers to periodically sift through the gigantic amounts of information without use of a recommendation system based on algorithmic models, despite their potentially harmful consequences for privacy and associated rights (Eskens, 2020, p. 1124). More recently, states such as California (e.g. with the California’s Consumer Privacy Act or the California Privacy Rights Act) seem to incorporate some elements of the principle-based and largely technology-neutral data protection law that we discuss in the subsequent section (Chander et al., 2021). This and similar developments suggest that the divergence between the US and European countries that arose after the establishment of the FIPPs in the 1960s might be decreasing once again (Hoofnagle et al., 2019, p. 70; Rustad & Koenig, 2019, pp. 387–405).

#### Data Protection

In the context of datafication, and especially given the intensification of data creation and circulation, and the increased use of large-scale computing devices from the 1960s onwards, there was growing attention for the regulation of such processes. The demand for transparency increased, and with it, the duty to inform an individual upon request whether one is subject to data processing, what use is made of personal data, who has access to it, and for what reason (van der Sloot, 2014, pp. 314–316). As the individual became the ‘data subject’, concerns were articulated around transparent and accountable collection, storage and processing of personal data. As long as highly specialised and expensive machinery was used, concerns around the use of computerised data were closely tied to specific purposes. Any collection and processing of data had to be carefully considered and planned, since only very limited resources for collection, processing and storage were available. Therefore, similarly to what has been described in a US-American context in the previous section, regulation addressed the use of data predominantly by sector. Governance frameworks were developed with specific applications in mind and with the aim of becoming the basis for specific ‘hands-on’ management practices.

During the 1970s, many European countries further reacted to the technological developments that were taking place. While they were adopting some rules established in the USA—in particular the FIPPs—the European type of regulation was no longer limited to specific sectors. Rather, the principle-based and technology neutral ‘omnibus approach’ emerged (Hoofnagle et al., 2019, pp. 69–72). The development of this ‘data protection law’ must be considered in parallel with the development of frameworks based on the protection of privacy as an individual right. The first data protection frameworks came into force in Germany (first regional law in Hesse in 1970) and Sweden (worldwide first national data protection law from 1973; Gstrein, 2016, pp. 43–44). Given the relatively large number of European countries, there was a need for harmonisation to ensure compatibility of national regulatory frameworks. Hence, the Council of European—an intergovernmental organisation with its seat in Strasbourg, France—developed a Convention for the protection of individuals with regard to the automated processing of personal data (Convention 108), adopted in 1981. This first and only legally binding international treaty on data protection law was revised in May 2018 and is open for non-members as well (Council of Europe, 2018). Proponents of the modernised convention hope that it will become the nucleus of more multilateral approaches to regulation and governance in the area (Kwasny et al., 2020). Convention 108 + contains several key principles to be applied while collecting, storing and processing personal data. These include lawfulness, fairness, transparency, purpose limitation, data minimisation, accuracy, storage limitation, integrity and confidentiality, as well as accountability (Ukrow, 2018, p. 243). While these principles mirror much of the FIPPs, they are worked out in more detail including associated individual rights and duties for those collecting and benefitting from the use of PII.

It goes beyond the scope of this article to discuss all of these in detail, but it is important to highlight that the omnibus-style data protection regulation also increased attention for individual rights and remedies. These typically include the right to be informed about data collection and processing, to obtain confirmation of processing, to restrict and object to processing, to rectify personal data, or to erase it. The European Union General Data Protection Regulation (GDPR) from 2016—probably the most prominent offspring of Convention 108 to date—includes further rights. Among these are the right to de-list entries from the index of a search engine (delisting, or somewhat inaccurately called a ‘right to be forgotten’), a right to data portability (e.g. moving personal data easily between different internet services), as well as a right not to be subject to decisions that have been taken solely through automated means (AI, Machine Learning, etc.; Ukrow, 2018, p. 245). The GDPR strongly emphasises privacy and data protection as two intertwined yet fully independent human rights. This understanding gained traction with the adoption of a Charter of Fundamental Rights by the EU in the early 2000s, which became legally binding with the treaty of Lisbon that came into force on 1 December 2009 and contains both a right to the protection of privacy in Article 7 and data protection in Article 8 (González Fuster, 2014).

Data protection has emerged as one of the mainstream approaches to regulate the protection of privacy. By 2020, most countries worldwide had specific data protection legislation. With 62 new data protection laws coming into force in the period spanning 2010 to 2019, their overall number had risen to 142 by the end of the decade. Most of these are omnibus style frameworks, which is particularly noteworthy since they are mostly applied outside Europe or the USA (Greenleaf & Cottier, 2020). The COVID-19 pandemic might have delayed further development of legal frameworks, but their number has continued to grow to 145 in 2021 with the influence of GDPR-style regulation increasing, as states revise older frameworks accordingly (Greenleaf, 2021). Arguably, as datafication becomes more and more prominent and deployed in increasingly generic ways across spheres of activity, it also becomes more difficult to address new developments with sector-specific rules. This is especially challenging for large democracies, where it can be difficult to create momentum to regulate a specific data-driven practice. This tendency further stresses the importance of understanding of datafication as a process, and the value of the framework provided in Sect. 3. While sector-specific rules come with the advantages that they are more concrete and practice-oriented, omnibus frameworks cover more practices, establish comparatively clear rights and duties, and manifest an approach that emphasises the dignity of the individual.

Nevertheless, the abstract nature of the omnibus approach results in the need to clearly define concepts such as personal data, which remains a challenge both on the legal (Finck & Pallas, 2020; Gstrein & Ritsema van Eck, 2018, pp. 80–81) and philosophical level (Søe, 2021). Purtova highlights the risk that the increasing datafication of society combined with the extensive scope of European data protection law might eventually make data protection ‘the law of everything’ (Purtova, 2018, p. 41). At the same time, data protection regimes require independent, strong and adequately resourced data protection authorities to interpret and effectively enforce the provisions, as well as independent courts capable of safeguarding the rule of law. A final point of concern is the strong focus of data protection on the individual, which misses the collective dimension of data-driven practices. We will return to this at a later stage.

#### Habeas data

We have already stressed that privacy should not be understood as an isolated concept, but rather as a proxy for the relationship between the individual and society. In this sense, the meaning of privacy evolves along the vectors of time, space and culture. While Cannataci argues that culture can be sub-divided in fields such as economic and technological development (Cannataci, 2016, pp. 8–10), the importance of political processes should not be underestimated. Few concepts relating to privacy and the control of information flows demonstrate this as clearly as habeas data, since its emergence is closely tied to the political history of countries in Latin America throughout the twentieth century (Gonzalez, 2015, p. 649). The expression ‘habeas data’ is derived from the more prominent legal principle ‘habeas corpus’, which is Latin for ‘you should have the body’. In common law jurisdictions, this legal notion—or ‘writ’—describes the requirement to bring a prisoner or detainee physically before the court to decide whether detention is lawful. In analogy, habeas data require public authorities to provide all personal data relating to a certain case or allegation to ensure that a person is aware of the basis of the accusations they are confronted with (Parraguez Kobek & Caldera, 2016, p. 114). Such a requirement for full transparency should enable the individual to understand the situation, correct false statements and rectify data. However, the dimensions and implementations of the concept differ among the eleven Latin American countries that have it enshrined in their constitutions (Parraguez Kobek & Caldera, 2016, 116). This makes it difficult to precisely define habeas data as a concept. Narrowly understood, it is an individual right that was developed in response to military dictatorship. It entitles a citizen to obtain information about oneself or close family members only. More broadly understood, it enables anyone to access information from public archives and potentially also from private sources in cases where this information might be of public interest (Gonzalez, 2015, p. 649).

While the Brazilian constitution from 1988 might include the first formal manifestation of habeas data (Article 5, section LXXII), countries such as Colombia, Peru, Argentina, and Venezuela also put similar—but not identical—provisions in their constitutions, adopted throughout the 1990s (Gonzalez, 2015, pp. 651–658). Furthermore, it is difficult to establish whether habeas data have its roots in traditional privacy provisions or data protection frameworks such as Convention 108. Since both are mentioned by scholars, one might conclude the answer lies somewhere in-between (Gonzalez, 2015, pp. 651–653; Parraguez Kobek & Caldera, 2016, pp. 113–114). Finally, in times of increasing use of automated decision-making and self-learning algorithms exploiting Big Data, one might speculate whether a progressive interpretation focused on the primacy of regulation could transform habeas data into a response to the threat of a ‘Black Box Society’ (Pasquale, 2015), where the actual grounds or data for decisions affecting individuals are hardly retrievable. To the contrary, if one believes that explainable AI/machine learning is not realistic and the benefits of rapid technological advancements outweigh potential concerns around trustworthiness, such an interpretation of habeas data would be understood as misguided and unrealistic. As such, a transformation of the concept of habeas data potentially cuts across the four layers of our datafication model, as a wholly comprehensive principle.

Beyond these speculations, however, under-developed substantive concepts are not the stumbling block to effective protection of the informational sphere of individuals and groups in the region. To illustrate this with one example: Latin America’s largest country Brazil has a comprehensive set of data protection and privacy frameworks including a dedicated Internet Bill of Rights (‘Marco Civil da Internet’), as well as a modern and comprehensive data protection law that is inspired by GDPR (Parentoni & Souza Lima, 2019; Souza et al., 2017; Viola de Azevedo Cunha & Itagiba, 2016, pp. 635–636). While it might sometimes be challenging to enforce these frameworks since their provisions are partly contradictory (Parentoni & Souza Lima, 2019, 6–14), the main problem is the costly and complex investigations that have to be carried out by independent enforcement authorities. Shortcomings in institutional effectiveness are not only an issue in Brazil or Latin America (Ryan, 2020), but in this context they do undermine the usefulness, appropriateness and efficiency of habeas data.

#### Informational self-determination

Many governments run periodic large-scale census programs to understand how their populations live and to project how they might develop in the future. These data contribute to planning decisions and to the development of policies. However, such drive to collect personal and household data keeps sparking concerns about the relationship between citizens and governments. Australia had a public debate on the granularity of census data collection in 2016 (Galloway, 2016), and in the USA there was a discussion on whether citizenship should be registered during the planned 2020 census (Adelman, 2018, pp. 550–551). In response to a similar situation in Germany that led to a legal conflict, the (West-) German Federal Constitutional Court (FCC) proclaimed the constitutional right to ‘informational self-determination’ on 15 December 1983 (Brink, 2017, p. 433).

This individual right is noteworthy for several reasons. First, it was developed by the judges through combining provisions enshrined in the first two articles of the German Basic Law (‘Grundgesetz’) that came into effect in 1949. Based on the normative statements that ‘human dignity shall be inviolable’ enshrined in Article 1 paragraph 1, as well as that ‘every person shall have the right to free development of his [sic] personality’ enshrined in Article 2 paragraph 1 of the Basic Law, the court decided that such space of personal discretion must also exist in the informational sphere. Hence, informational self-determination is a specific and independent individual right that is not based on traditional privacy provisions and has no direct connection to data protection, or the general right to personal development. In the concrete context of this decision, it means that a citizen must not be afraid of what the state *could* know through the collection of data (Brink, 2017, p. 433). Sometimes this is also described with the term ‘chilling effect’, which means that citizens (and particularly journalists) should not refrain from certain behaviour or statements for fear of retaliation by state authorities (Bradshaw, 2017, pp. 347–348). This concern is directly tied to the reflexive dimensions of datafication, where looping effects between the different elements of datafication have an important effect.

Secondly, the right to informational self-determination has been remodelled over the decades. It has not only become applicable to some information held by security services in East-Germany (Walther, 2016), but the FCC has also explored its application in a Digital Age dominated by the forces of ‘surveillance capitalism’ (Zuboff, 2019). In two decisions from 2019 on the ‘right to be forgotten’, the FCC arguably expanded informational self-determination to also include commercial exploitation of data by technology giants, insofar as they limit the space of individual discretion and informational self-determination (Gstrein, 2020b, pp. 136–139).

This judgement extends informational self-determination to a context that is limited to private parties. At this point in time, it is still too early to know what this will mean, especially since human and civil rights typically remain limited to the relationship between states and citizens when it comes to enforcement (van der Walt, 2014, pp. 201–203). Furthermore, it remains to be seen whether the courts will uphold this line of interpretation of informational self-determination as also applying to corporate contexts, and repeat or even expand this stance in future judgements. Nevertheless, it is an interesting line of jurisprudence that could place the discourse on the collection, control and use of personal data in a new perspective. This brings us to a third reason why this concept is interesting. The right to informational self-determination might be important to make progress on issues of identity management, especially in situations, where the increased use of social networks requires society to develop something like ‘post-mortem privacy’ for accounts with deceased controllers (Buitelaar, 2017, p. 137), or when exploring the regulation of novel forms of digital identity management along the notion of the ‘self-sovereign individual’ (Zwitter et al., 2020).

In conclusion, it seems that informational self-determination as a concept still has potential and is likely to be further elaborated. Furthermore, one might argue that the concept can also be easily transferred on the level of the European Union, since Article 1 of the Charter of Fundamental Rights of the EU also includes a legally binding commitment to human dignity that is practically identical to the German Basic Law. However, critics of the concept argue that it has a utopian and almost metaphysical character, which makes informational self-determination impractical in terms of execution and enforcement (Veil, 2018, pp. 687–688). It remains to be seen whether a potential Europeanisation or internationalisation of the concept will eventually make it more relevant, detailed and future-proof for settings beyond Germany. One of the avenues to achieve this might be associated with a refined understanding of human dignity. In the context of privacy and datafication, human dignity might be understood as the ability to legitimately exist as human ‘with twists and turns’ and to have the ability to create shared spaces for discretion with partners and groups (Floridi, 2016).

### Emerging conceptual approaches

In this section we augment the analysis of contrasting legal approaches to the protection of privacy with three recently emerged approaches that aim to address the consequences of datafication.

#### Differential privacy

Like informational self-determination, the emergence of differential privacy (DP) is tied to the creation of statistics and the problem of how to gain insights on populations in a responsible manner. While the essential problem—identifying trends in the development of a population with safeguards for individual privacy and autonomy—remains the same, DP uses mathematics and statistics to enable ‘protection by default’ (CACM Staff, 2021). The term was first used in 2006, but the concept gradually emerged around 2003, building on earlier research that addressed the issue of how to produce privacy-preserving statistics (Dwork et al., 2017, pp. 17; 20–22; 29; Le Ny, 2020, pp. 9–11). More recently DP has gained the attention of large technology companies that try to apply it in the context of market research and for the training of algorithms, as well as the development of apps and services. It also seems appealing to public institutions such as the US Census Bureau (CACM Staff, 2021, p. 36).

The underlying problem that DP aims to address is that any large dataset does not only allow one to retrieve information on the entire group, but also the individuals that are included in it. DP should enable the generation of insights and trends based on the entire dataset and make it difficult to infer characteristics relating to individuals. In other words, ‘what differential privacy aims for is not to prevent information disclosure per se but to guarantee that, if an individual provides her data, it does not become significantly easier to make new inferences about that specific individual compared to the situation where her record is not in the dataset’ (Le Ny, 2020, p. 4). This is achieved by manipulating the dataset after collection. Any search query against a dataset ‘is perturbed by the addition of random noise generated according to a carefully chosen distribution, and this response, the true answer plus noise, is returned to the user’ (Dwork et al., 2017, p. 17). Hence, the analyst of the dataset does not get an entirely accurate result to a search query but is informed about significant trends and tendencies that exist throughout the set. There are three important parameters that need to be considered: the accuracy of the data as initially collected, the addition of noise to manipulate the set and conceal characteristics of individuals, as well as the number of queries available to protect individuals and keep the dataset useful (often called the ‘privacy budget’).

On the one hand, the strengths of this approach lie in the protection of privacy by default. It might potentially enable the responsible use of very large datasets, without limiting individual autonomy. This could be especially beneficial in scenarios where large datasets are needed to train algorithms and autonomous systems. It also remains to be seen whether DP could be merged with the creation of ‘synthetic data’, which is another very recent trend where data are modelled after other ‘real data’, collected from the observation of individuals. This additional synthetic data can be used to augment the training processes of artificial intelligence (Hao, 2021; Zhang et al., 2019, pp. 1837–1838). Synthetic data have the advantages that it is very accurate, complete and not private. Nevertheless, questions of appropriate or fair representation of different strata and groups of society will certainly spark discussions on responsible and ethical use of synthetic data.

On the other hand, DP seems difficult to implement in practice since there needs to be sustained awareness of the fundamental parameters necessary to consistently apply the method and keep the dataset useful. Furthermore, the interpretation of datasets that have been manipulated according to the approach require a high amount of data literacy to draw useful conclusions (CACM Staff, 2021, pp. 40–43). At the same time, the use of DP will not contribute to alleviating privacy-related concerns for applications where it is necessary to have very accurate individual data. Finally, while individual autonomy might be protected with the implementation of DP in large datasets, relevant insights about the entire group are still readily available. Therefore, a further limitation is that the question of collective autonomy cannot be addressed with this approach. In addition, this approach to data contrasts strongly with the data mining spirit that supports much of datafication, in which intuitive exploration of ever larger datasets dominates—rather than the disciplined questioning of restricted data prescribed by differential privacy.

#### Contextual Integrity

Datafication has intensified in the last two decades with the advent of increasing amounts of data, created and shared using improved connectivity infrastructure, as well as our growing engagement with mobile devices for work and leisure. This has certainly created new opportunities for the development of smarter algorithms and more autonomous systems (Furht & Villanustre, 2016, pp. 3–12). Nevertheless, changing patterns of data creation, use and circulation have made it more challenging to define the purpose of data collection and use from the outset—two important elements to implement privacy-enhancing measures. This means that traditional management strategies such as notice and consent have become increasingly difficult (perhaps even meaningless) to apply (Eskens, 2020, pp. 1117–1118). This is particularly challenging for European approaches such as the GDPR, where the use and collection of data are only possible if a legal basis has been established. Without such a legal basis, personal data are not allowed to ‘flow’ at all. This can seem like an artificial requirement that is not in line with societal reality in the digital age.

Contextual integrity (CI) is an approach that might offer an alternative to the difficulties of the notice and consent requirement: ‘According to the theory of contextual integrity (CI), privacy, defined as CI, is preserved when information flows generated by an action or practice conform to legitimate contextual informational norms; it is violated when they are breached’ (Nissenbaum, 2019, p. 224). CI was first proposed in 2010 and does not focus on the rights and duties of individuals. Helen Nissenbaum’s theory can be described along four theses that build on each other (Nissenbaum, 2019, pp. 225–234):

1. Privacy is the appropriate flow of personal information.
2. Appropriate flows conform with CI Norms (‘Privacy Norms’).
3. Five parameters define privacy (CI) norms: subject, sender, recipient, information type and transmission principle.
4. The ethical legitimacy of privacy norms is evaluated in terms of: A) Interests of affected parties, B) Ethical and political values and C) Contextual functions, purposes and values.

CI originates from a reinterpretation of the established US concept of a ‘reasonable expectation of privacy’ (Nissenbaum, 2011, p. 32). Rather than highlighting transparency and choice, which seems to be increasingly challenged in datafied environments, the context and integrity of data generation, processing, storage and sharing become central. CI is articulated according to the reasonable expectations of privacy of a data subject in a particular context. This means that expectations about sharing name and address might be very different in the context of a customer loyalty program versus sharing the same data for a medical consultation. CI requires more or less safeguards and privacy preserving mechanisms, depending on the context. In other words, privacy becomes a matter of integrity and compliance with social norms. On the one hand, this results in a responsibility for each single party involved in the use of personal data to comply with such social norms. On the other hand, the adoption of this logic might result in a dynamic that increases the influence of those who disproportionately benefit from the use of personal data and who have more resources to shape the use and collection of PII. Therefore, an uncritical adoption of CI might allow such powerful actors to promote one-size-fits-all solutions that lower existing privacy protection standards in some areas of the world (Wu et al., 2020, pp. 486–488). Whether or not this seems acceptable and desirable also depends on cultural expectations and traditions. For instance, in settings where the African philosophy of Ubuntu applies, CI in and for groups might be more important than individual concerns around the use of PII and high levels of protection from an individual perspective (Reviglio & Alunge, 2020, pp. 8–15).

While CI focuses on context to establish the significance for privacy, it could also be connected to the work of Søe, who adds a distinction between natural (‘information that’) and non-natural information (‘information about’) to context, building on the language philosophy of Grice (1967), and Scarantino and Piccinini (2010). This stance avoids a complex discussion of what makes data personal in order to protect privacy. Søe states that ‘[i]nstead of speaking of personal information as digital footprints with the objective neutral understanding lying underneath, we can start speaking of personal information as something that changes meaning when shifted from one context to another; as something that might become misleading if other information is added; and as something that is already in the realm of communication, convention, and interpretation—particularly when it is derived from people’s actions in the online domain (i.e., clicks, likes, browser history)’ (Søe, 2021, p. 2).

Such consideration of the contextual transformation seems to be appropriate since many existing interpretations of the right to privacy are already tailored to specific contexts like criminal procedures (Selbst, 2013, pp. 699–705). Furthermore, over-burdened individuals might rather enjoy datafication in contexts that have been designed along principles of ‘privacy by design’ and ‘privacy by default’, since this would include corresponding information security elements (Orrù, 2017, pp. 107–111; Wu et al., 2020, p. 488). Nevertheless, while consent might not be an ideal tool to exercise control, it serves as a persistent reminder of how difficult it has become to exercise agency. A switch towards a concept that focuses more on integrity than on individual rights, may background the autonomy and dignity of the individual as required by international human rights law. In other words, this could lead to a trade-off between protection offered by enforcing contextual integrity and high expectations of autonomy and individual choice.

Another set of issues arises as we consider the entwinement of contexts so that they become contiguous in digital and networked settings (Beaulieu & Estalella, 2012) and the increasing flow between contexts that datafication often brings about. For instance, how will different assumptions of CI be managed in international data flows and can we navigate between them? Instead of increasing the space for individual discretion, this ‘normalisation’ of data management may tend towards ‘one size fits all’ approaches as already mentioned. It may be more difficult for an individual to insist on personal choice and individual rights if there is disagreement with the majority (in a democratic system), or powerful forces (in any other political or economic system). Therefore, CI is promising from a policy perspective that presumes high ethical and moral standards, but it remains to be seen how concrete duties and responsibilities can be established and enforced.

#### Group Privacy

The final approach considered in more detail also aims to respond to the changing realities of datafication. Many harms caused by these new practices, especially around the use of algorithmically driven systems, are not directly relatable to personal data or to a data subject. Usually, such individual source data are first aggregated, then analysed and re-interpreted before being put into action (van der Sloot, 2017, pp. 215–223). A good example to illustrate this is predictive policing, where historic crime data are merged with many other data sources to project the likelihood of future crime in a neighbourhood or among a social group. Although it is difficult to link the projections of the system to individual data at the source, it is clear that the use of predictive policing systems has tangible consequences for both individuals and groups (see for instance the context of Chicago as described by Stroud, 2021). Hence, we need to discuss both how existing legal frameworks fail to cover many aspects of the use of predictive policing and the self-learning algorithms at their core, and how ethical and societal factors influence it (Gstrein et al., 2019, pp. 93–94). This scholarship can further be connected with the changing nature of public space in cities that are constantly being monitored and managed through information technology (Boanada-Fuchs, 2018, pp. 52–55)—a prime example of datafication.

Hence, scholars such as Floridi have started to consider group privacy in order to overcome ethical approaches that are either too focused on natural persons or atomistic agents (Floridi, 2014). The basic idea is that groups that are affected by datafication respond by organising themselves and becoming a relevant player with a stake in the decision-making processes. This approach is still relatively young and mostly limited to the academic sphere, though there are grass-roots social movements that embrace this approach and address the issues of data, infrastructure and access at group and community level (e.g. Detroit Community Technology Project, 2021; Our Data Bodies Project, 2021).

The idea is that if rights are articulated at group level, this might help address regulatory shortcomings of individual rights that fail to enable persons to develop themselves and shape a dignified existence (van der Sloot, 2017, pp. 222–223). However, there are many challenges when trying to operationalise group privacy. First, it is difficult to understand how such a group should be constituted. Groups have been described along the categories real/fictional, self-proclaimed/framed, self-aware/not, stable/fluid, as well as hierarchical/egalitarian (Taylor et al., 2017, pp. 227–231). Without going into detail on all of these, groups are often unaware that they are treated as such, since they can be generated almost arbitrarily through the structuring of datasets. When deciding whom to ‘target’ for an advertising campaign on a social media platform, the ordering party has a rich choice of parameters including age, gender, location, intensity of use of the platform, types of contributions, etc. The resulting ‘target group’ will not necessarily be aware of its existence, since it has little or no influence on its composition. However, it is the case that such groupings often reproduce socio-economic and cultural hierarchies. Returning to our example of predictive policing, data used for crime prediction may further embed institutional racism since victims and perpetrators will be over-represented in historic datasets on which algorithms are trained. The datafication of groups might thereby add yet another instance to existing inequalities. Furthermore, there is the question whether and how such groups could be organised or organise themselves. Some groups might have a hierarchical structure, others less so. Finally, even if a group is aware of its existence, or already recognised as vulnerable (e.g. minorities), how could its rights be integrated effectively in decision-making processes? There are already some emerging exemplars of how to organise such representation of groups as stakeholders, and of mechanisms to ensure representation and the responsible use of categories—ranging from establishing review boards or increasing team diversity to ensuring formal auditing and appeal processes (D’Ignazio & Klein, 2020).

## Discussion: Strengths, limitations and remaining issues around the approaches to privacy

In this article, we reviewed and contrasted various approaches to identify how to protect privacy in a datafied society. We have done so with the objective of identifying common denominators, overarching themes and important differences. Our overview has explored many of the entwinements of technologies, regulatory frameworks, and public and corporate values. Following up on our careful mapping of key concepts, we move on to point to further trends where privacy and datafication intersect. While we have stressed diversity and plurality, not all of the entwinements are equally prominent. In this section, we suggest particular alignments that deserve attention and solidify. Consideration of these alignments might be useful to stimulate further thinking when seeking answers on how to protect privacy in a datafied society. More specifically, we hope that our work can serve as a starting point to identify potential synergies and friction between the different approaches we presented. Across the various themes, we note how each of the alignments seem to support or challenge privacy in each case.

### Data subjects and personalisation

The interaction between datafication and privacy is especially visible in relation to the emergence of the data subject. The necessity to define and ‘protect it’ has been strongly developed by legal frameworks such as the European Union’s Charter of Fundamental Rights and the GDPR (González Fuster, 2014). The role of the data subject is set in contrast to the role of the ‘controller’ of the data operation, as well as the ‘processor’ that supports the controller with technical and organisational capabilities. We have discussed the importance of datafication for a growing range of activities that define social, material and intellectual relationships. Having friends, getting a bank account or pursuing education have all become activities that can hardly take place without a wealth of digital data and traces being generated and collected. To be recognised as a person—whether as friend, customer or student—increasingly depends on the production, validation and circulation of digital data.

The role of physical distinctions in articulating privacy has also diminished. Privacy cannot be established and protected based on a physical space that cannot be entered. It has become increasingly difficult to uphold privacy protection as being about boundaries that must be maintained, and within which a person cannot be known, and no data should be captured. Using the argument of personalisation, many platforms have eroded the boundaries between what would be private, or what would belong in different individual or collective spheres, by increasingly combining and circulating personal data with the justification of providing more personalised services to customers. Extreme personalisation may be decreasing privacy. First, it makes the personal public through greater data circulation, and second, it personalises what were considered public spheres (care, education, political engagement), while at the same time relying on types, classes and categories on which recommender algorithms are built (Lury & Day, 2019). To illustrate this complex dynamic: there is currently no way of knowing how ‘liking’ pictures of a baby of a friend on a social media platform will also affect the kinds of job ads brought to the attention of someone or even influence a hiring process that includes how candidates use social media.

Additionally, omnipresent sensors and digital infrastructure lead to ‘technological gentrification’, where the choices of those who individually resist becoming part of the data society are rendered meaningless (Gstrein & Ritsema van Eck, 2018, p. 76). Hence, privacy becomes an issue of circulation, combination and evaluation of data, rather than of labelling data for a specifically definable and static purpose, which is typical for data protection law. The correspondence of personalisation and of privacy as associated with a subject is not accidental: these dynamics share deep roots in systems of ownership and rights. Privacy is then not tied to a special place, but to the possibility for the data subject to modulate, be informed, and keep control on what one allows to circulate, to whom and for what purpose. Nevertheless, to support the data subject in exerting their privacy, there needs to be accountability and transparency around actors, data sharing and data circulation and erasure, as well as means of recourse when something goes wrong with one of those aspects.

### Context and privacy: The public and the corporate

The circulation of data challenges the boundaries between citizens and consumers as more and more public services come to rely on platforms provided by corporations. For example, digital contact tracing apps have been deployed in response to the COVID-19 crisis, so that public health is being entwined with commercial services, raising concerns both about individual and collective autonomy/sovereignty (Veale, 2020). This creates tensions between our understandings of what kind of privacy can be expected in different contexts, since expectations around privacy differ between regions of the world and between purposes. For example, the GDPR requires that special categories of data—such as health data—be treated with special safeguards enshrined in Article 9. The general rule here, however, is that whenever possible, such data should not be used. To support privacy from this approach, clarity about the identity of the actors is crucial, and sharper boundaries between types of uses need to be maintained. Next to the importance of having transparency around the actors and their roles, it also seems that the principle of purpose limitation plays a crucial role in specifying the context of use. While strict purpose limitation can affect the promises associated with Big Data and algorithmic pattern recognition, it helps to provide essential criteria for the protection of fundamental rights such as legitimate aim, legality and proportionality (Koning, 2020, pp. 231–232).

### Data protection: consent, the individual and the collective

Data protection frameworks have the advantage of clearly defined roles, which can then be associated with concrete individual rights and duties. However, data protection is an abstract policy objective. When framed as a fundamental right such as in Article 8 CFEU, its purpose and legitimacy remain implicit. Furthermore, this approach has a static view of data. To legally assess and qualify data during collection, processing and analysis, we need to use labels such as private/non-private, general/special or appropriate legal basis. Building on the latter, the limits of such a static and formal approach becomes visible when discussing the nature of consent, a concept further defined in the development of GDPR (de Hert & Papakonstantinou, 2016, pp. 187–188). The necessity to gain individual consent for most uses of PII seems to put the individual in a strong position. Recall that consent needs to be freely given, and to be specific and informed, and to constitute an unambiguous indication of one’s wishes. Nevertheless, the sheer number of data-driven applications requiring consent has exploded in the context of datafication, so that the feasibility of consent is a very real issue. In addition, these applications and the business models behind them are often built according to a logic of circulation. As a result, the free flow and combination of data become the default that serves the interests of these corporate actors, and users of systems find it difficult to opt-out or withhold consent because the consequence is exclusion from services that have become essential.

In addition, the possibility of drawing inferences on individual behaviour and characteristics using state-of-the art data analytics raises the question whether the focus on the individual is appropriate in the first place. In other words, data analytics can generate profiles that are precise enough to identify individuals—and therefore breach privacy—and can do this based on data that does not fall under the category of PII and does not require consent. Certainly, consent manifests the important role of individual autonomy and human dignity, but it often fails when it comes to practical and efficient enforcement.

## Conclusion

In this article, we focused on exploring the intersection of datafication and the protection of privacy. The approaches and the exemplars we have presented are a useful heuristic for navigating complex developments. Of course, none of these are islands, certainly not with the connecting impulse of the digital sphere. Our mapping and discussion of these different approaches are a useful contribution to understanding differences and the relevance of social and institutional circumstances for their proper functioning. Furthermore, the dynamics we have described are deserving of much more attention and suggest a series of important lines of inquiry: How do encounters between these various versions of privacy and personhood fare? Is there an ‘interoperability’ of privacy at work as data increasingly circulate globally? Is it the case that several different approaches thrive or are we moving towards a monoculture of privacy focused on the least common denominator? Indeed, is it necessary that only one of the presented approaches emerges as the single solution to the complex challenges? It seems that some of them will work well in different societal circumstances and disciplinary contexts. For instance, data protection might be a useful approach from a legal perspective, whereas approaches such as habeas data and informational self-determination might be especially strong when it comes to establishing governance principles and political guidelines. DP could become a useful standard tool for the creation of privacy-preserving statistics, whereas CI could become a guiding ethos for the design of computer and information sharing systems that focus on high levels of information security. Finally, group privacy might be an approach that has its merits in the context of social sciences and the development of ethical frameworks for the use of PII. Rather than trying to find one single solution that works across all these settings and worldwide, it might be more useful to identify synergies and stumbling blocks to make sure privacy is respected, protected and promoted where it is necessary and desirable.

To conclude, we want to stress the variety of configurations of cultural, institutional, technological and political elements that gave rise to the various conceptualisations of privacy analysed. If the right to privacy was part of nation building in America, the doctrine of habeas data was not less politically fuelled. If informational self-determination was considered an essential part of self-realisation, then the right to be forgotten by search engines is a fascinating articulation of this need. And if data protection also started as a right focused on individuals, it has since been reframed to insist on transparency and accountability of organisations. It also seems that some approaches to privacy may be less amenable to certain technological configurations. For example, habeas data would not be easily reconcilable with forms of AI that are not explainable. Similarly, the informational self-determination approach has many points of friction with the consent and licensing approach, or ‘data ownership’ by parties the personal information does not directly refer to. As new social and technological forms develop in the digital age, it is important to be aware of such enhancing and limiting characteristics. Nevertheless, any commitment to the alleviation of privacy problems also prompts a pragmatic and targeted set of measures. With this article, we hope to contribute to a better understanding of the broad and rich basis of approaches that are available to design such urgently needed measures carefully, effectively and diligently.
